# CALENDAR

**DOI:** 10.1054/bjoc.2001.1780

**Published:** 2001-04


					29 November - 1 December 2001
3rd Biennial International Meeting of the Flemish
Gynaecological Oncology Group (FGOG)
KBC Building, Brussels, Belgium
Topics:
* ER Research
* Endocrine Prevention of Gynaecological and Breast Cancers
* Adjuvant Endocrine Therapy in Gynaecological and Breast
Cancers
* Endocrine Therapy in Metastatic Breast Disease
* The Uterine Effects of SERMS
* Oestrogens and the breast (cancer)
* Progesterons and Oestrogen - Exposed endometrium
* HRT following gynaecological and breast cancer
Further information from:
Angele Segers, University Hospitals Leuven, Gynaecologic
Oncology, 3000 Leuven, Belgium. Tel: + 32 478 59 83 80;
Fax: +32 16 34 76 87; E-mail:vwog2001@yahoo.com;
Website: www.kuleuven.ac.be/vwog2001
Errata
Br J Cancer 84(3): 423-428
Predicted long-term mortality reduction associated with the
second round of breast screening in East Anglia
J McCann, S Duffy and N Day on behalf of the East Anglian
breast screening programme (Director of Quality Assurance:
R Warren).
In the Discussion section, the second sentence of the second para-
graph should read: `These predictions may underestimate the true
impact of screening, since the 3 years' lead time allowed to adjust
for the earlier diagnoses of screen detected cancers in the invited
group may be an overestimate.
Br J Cancer 84(1): 141-146
Childhood cancer and parental use of tobacco: findings from the
inter-regional epidemiological study of childhood cancer
(IRESCC)
T Sorahan, PA McKinney, JR Mann, RJ Lancashire, CA Stiller,
JM Birch, HE Dodd and RA Cartwright
Table 1 contained one inccorect line of data; the first line of data
in the Mothers section should have been deleted. The publishers
apologize for this error and the Table is reproduced correctly
below:
CALENDAR
British Journal of Cancer (2001) 84(7), 1010
(c) 2001 Cancer Research Campaign
doi: 10.1054/ bjoc.2000.1780, available online at http://www.idealibrary.com on
1010
Table 1 Childhood cancer risks by parental cigarette smoking habits before the pregnancy (time of conception): IRESCC data, 1980-1983 diagnoses
Childhood cancer risk Mean birthweight (ounces)
Parental smoking Cases GP Hospital Cases vs GP controls Cases vs Hospital controls Cases GP Hospital
habit controls controls controls controls
(n) (n) (n) RR 95% CI RR 95% CI
Fathers
Lifelong non-smoker 184 218 171 1.0 1.0 115.9 119.3 117.2
< 10 cpd 26 34 27 0.94 (0.53-1.66) 0.92 (0.51-1.65) 120.1 114.2 116.6
10-19 cpd 79 60 70 1.63a
(1.10-2.41) 1.06 (0.72-1.56) 114.8 115.1 113.6
20-29 cpd 143 122 121 1.46a
(1.05-2.03) 1.11 (0.80-1.53) 118.1 116.9 117.0
30-39 cpd 23 32 48 0.95 (0.52-1.73) 0.45(a)
(0.26-0.77) 117.0 118.7 119.4
 40 cpd 28 21 40 1.77 (0.94-3.34) 0.66 (0.39-1.11) 117.0 109.2 113.6
P-value for trendc
P = 0.02 [P = 0.16]
Ex-smoker 43 51 47 0.99 (0.62-1.58) 0.90 (0.57-1.42) 121.7 119.0 119.4
Smoking status n/k 29 17 30 118.0 116.2 113.7
Total 555 555 554
Mothers
Lifelong non-smoker 283 316 234 1.0 1.0 118.9 118.7 118.8
< 10 cpd 46 30 43 1.77a
(1.07-2.92) 0.87 (0.54-1.39) 119.2 114.4 121.5
10-19 cpd 114 88 100 1.51a
(1.08-2.13) 0.95 (0.69-1.31) 115.1 114.0 113.4
20-29 cpd 78 74 103 1.22 (0.86-1.74) 0.64(a)
(0.45-0.91) 114.2 113.9 113.6
 30 cpd 7 14 36 0.48 (0.17-1.37) 0.18(b)
(0.08-0.40) 98.0 121.2 111.6
P-value for trendc
P = 0.53 [P < 0.001]
Ex-smoker 21 27 31 0.89 (0.49-1.62) 0.58 (0.32-1.05) 117.7 127.0 120.4
Total 549 549 547
a
P < 0.05; b
P < 0.001, () indicates deficit; c
two-tailed P-value, [] indicates negative trend; cpd = cigarettes per day

				

